# Care seeking for childhood illnesses in rural Mtwara, south-east Tanzania: a mixed methods study

**DOI:** 10.1093/trstmh/trae022

**Published:** 2024-05-03

**Authors:** Salum Mshamu, Judith Meta, Casiana Sanga, Nicholas Day, Mavuto Mukaka, Bipin Adhikari, Jacqueline Deen, Jakob Knudsen, Christopher Pell, Lorenz von Seidlein

**Affiliations:** CSK Research Solutions, Mtwara, Tanzania; Centre for Tropical Medicine and Global Health, Nuffield Department of Medicine, University of Oxford, Oxford, UK; Private Consultant, Social Scientist; CSK Research Solutions, Mtwara, Tanzania; Centre for Tropical Medicine and Global Health, Nuffield Department of Medicine, University of Oxford, Oxford, UK; Mahidol Oxford Tropical Medicine Research Unit, Faculty of Tropical Medicine, Mahidol University, Bangkok, Thailand; Centre for Tropical Medicine and Global Health, Nuffield Department of Medicine, University of Oxford, Oxford, UK; Mahidol Oxford Tropical Medicine Research Unit, Faculty of Tropical Medicine, Mahidol University, Bangkok, Thailand; Centre for Tropical Medicine and Global Health, Nuffield Department of Medicine, University of Oxford, Oxford, UK; Mahidol Oxford Tropical Medicine Research Unit, Faculty of Tropical Medicine, Mahidol University, Bangkok, Thailand; University of Philippines, Manila, Philippines; Royal Danish Academy – Architecture, Design, Conservation, Copenhagen, Denmark; Amsterdam University Medical Center, University of Amsterdam, Department of Global Health, Amsterdam, the Netherlands; Amsterdam Institute for Global Health and Development, Amsterdam, the Netherlands; Amsterdam Public Health Research Institute, Amsterdam, the Netherlands; Centre for Tropical Medicine and Global Health, Nuffield Department of Medicine, University of Oxford, Oxford, UK; Mahidol Oxford Tropical Medicine Research Unit, Faculty of Tropical Medicine, Mahidol University, Bangkok, Thailand

**Keywords:** child health, diarrhoea, health services, health services needs and demand, malaria, respiratory tract infections

## Abstract

**Background:**

Care seeking was assessed in preparation for a study of the health impact of novel design houses in rural Mtwara, Tanzania.

**Methods:**

A total of 578 residents of 60 villages participated in this mixed-methods study from April to August 2020. Among them, 550 participated in a healthcare-seeking survey, 17 in in-depth interviews and 28 in key informant interviews.

**Results:**

The decision to seek care was based on symptom severity (95.4% [370]). Caregivers first visited non-allopathic healthcare providers or were treated at home, which led to delays in seeking care at healthcare facilities. More than one-third (36.0% [140]) of respondents took >12 h seeking care at healthcare facilities. The majority (73.0% [282]) visited healthcare facilities, whereas around one-fifth (21.0% [80]) sought care at drug stores. Treatment costs deterred respondents from visiting healthcare facilities (61.4% [338]). Only 10 (3.6%) of the households surveyed reported that they were covered by health insurance.

**Conclusions:**

Quality of care, related to institutional factors, impacts timely care seeking for childhood illnesses in Mtwara, Tanzania. Ensuring accessibility of facilities is therefore not sufficient.

## Introduction

In sub-Saharan Africa, children aged <5 y (i.e. ‘under-five’) remain highly vulnerable to infectious diseases. Each year, malaria is responsible for about 247 million clinical cases and 619 000 malaria-related deaths occur worldwide.^1^ Diarrhoea accounts for >90% of under-five mortality in low- and middle-income countries.^2^ Acute lower respiratory tract infections contribute to approximately one-third of under-five mortality in sub-Saharan Africa.^3^

Although the United Nations’ Sustainable Development Goal 3 advocates for access to healthcare services for all, in sub-Saharan Africa, 17% of the population lives ≥2 h away from public health facilities.^4–6^ Inequities in access to healthcare in sub-Saharan Africa are exacerbated by the mismatch between a high disease burden and the lack of healthcare facilities in rural areas.^6^

Factors influencing decisions to seek care for childhood illnesses include personal factors, such as child age, sex, knowledge of caregiver and illness perceptions^4,7^; interpersonal and community factors, such as perceived quality of healthcare, influence from families, friends and social networks regarding healthcare; and institutional factors, such as access and affordability of healthcare.^7^ Seeking care is a complex process and, because of issues related to access and preference, caregivers may sometimes first visit non-allopathic providers or opt for home treatment,^8^ resulting in potential delays in seeking care.^9,10^

In preparation for the ‘Star Homes’ project, a randomised controlled trial of a novel design house intervention, a baseline assessment of healthcare-seeking behaviour for childhood illnesses, was conducted. The Star Homes project aims to evaluate the protective efficacy of a new house design targeting malaria, respiratory tract infections and diarrhoeal diseases in rural Tanzania.^11^ A total of 550 households from 60 villages are participating in this study, of which 110 have been allocated novel design houses, whereas the remaining 440 households reside in traditional wattle/daub houses as the control group.^11^ A baseline assessment of healthcare-seeking behaviour was needed to ensure the comprehensive capture of all disease episodes during the trial, particularly given the limited knowledge about healthcare seeking for childhood illnesses in Mtwara.

The main objective of this study was to explore the healthcare-seeking behaviour for childhood illnesses.

## Methods and setting

### Study location

Mtwara, located in south-eastern Tanzania, is a major cashew-producing area. More than 90% of its residents are involved in cashew farming. Subsistence farming and small-scale fishing are also important economic activities.^12^ Mtwara has a population of 1 634 947 across nine administrative district councils; this study was conducted in Mtwara District Council, which has a population of about 158 504 residing in 110 villages.^13^ There are 38 government healthcare facilities in Mtwara District Council.^14^

### Study design

This study utilised a mixed-methods approach, involving a questionnaire-based survey, interviews and focus group discussions (FGDs).

### Recruitment of study participants

A total of 578 residents of 60 villages participated in the study from April to August 2020. Among them, 550 participated in a healthcare-seeking survey, 17 in in-depth interviews (IDIs) and 28 in key informant interviews (KIIs). The number of interviews was determined by the point of data saturation. Parents/guardians of children aged <13 y participated in 14 FGDs each consisting of six to 10 participants (Table 1).

**Table 1.** Summary of qualitative data collection methods and a description of respondents (Mtwara District Council, April–August 2020)

Type of method	Number	Respondents’ descriptions
FGD	14 (6–10 participants each)	• At least one FGD per ward • Each FGD comprised participants from 3 to 5 villages • Each FGD had a balanced representation of male and female participants • Each participant had a child aged ≤13 y
IDI	17 (head of households participating in the Star Homes project)	• 8 from winners of Star Houses • 9 from comparison houses
KII	28 (key people in the community)	• 11 village chairpersons • 1 village executive officer • 11 healthcare facility staff • 5 community health workers

### Inclusion criteria

A participant had to be a household head recruited in the Star Homes project, aged ≥18 y and had to provide written informed consent. KIIs included selected influential community members, such as community leaders, community health workers and medical officers at local health facilities. For the FGDs, any parent/guardian from the Star Homes project who was taking care of a resident child aged <13 y was invited.

### Data collection

The questionnaire was developed to collect sociodemographic information of the participants and cues of action from a child's last illness episode, focusing on malaria, respiratory tract infections and diarrhoeal diseases. The questionnaire was pretested and piloted among 10 non-study participants. Some questions were revised after piloting and discussion with the research assistants. The KIIs and FGDs were conducted in Swahili in a quiet and private location using the interview guides. They were audio-recorded by trained research assistants. Written informed consent was obtained from all participants.

### Data management and analysis

Quantitative data were analysed using Stata software version 14 (Stata Corp, College Station, TX, USA) where categorical variables were analysed as proportions. Comparison tables were used to examine the differences in proportions where a χ^2^ test was used to assess the statistical significance of the differences. Medians and ranges were used to summarise continuous variables. Multivariable analysis was conducted to identify factors independently associated with healthcare-seeking behaviour. Statistical significance was ascertained when p<0.05. The 95% CIs are reported where appropriate.

Qualitative analysis was conducted using ATLAS.ti version 9 software (http://www.atlasti.com). Data were coded using a predeveloped codebook. The codebook was developed by the site investigators together with the research assistants. Data were coded by the research assistants familiar with the local sociocultural context. Coding was followed by thematic analysis, supported by extracting relevant quotations.

## Results

### Sociodemographic details

Among the 550 heads of households, 69.3% (381) were male and most were married (78.0% [429]). The majority of respondents relied on farming for their livelihood activities (95.8% [527]). Their monthly expenditure ranged from <50 000 (US$22) to >301 000Tsh (US$131) with 51.1% (281) at <50 000Tsh (US$22) (exchange rate: 1US$=2300Tsh [at June 2023]). Nearly one-half of the respondents did not possess any listed valuables (48.7% [268]). The illiteracy rate was 45.6% (251); 49.8% (274) had completed primary education, while 1.7% (nine) and 2.9% (16) had secondary and informal education, respectively. Two-thirds (66.1% [358]) of the study participants were aged <50 y (Table 2). Almost one-half (44.4% [244]) reported cooking indoors, mainly using firewood (99.8% [549]) (Table 2). Study participants did not have access to clean water and sanitation systems, more than one-half (57.6% [317]) were using surface water (from ponds, dams and lakes) for drinking and the majority (94.9% [522]) used pit latrines (Table 2).

**Table 2.** Respondents’ sociodemographic characteristics (Mtwara District Council, April–August 2020)

Respondents’ sociodemographic characteristics (n=550)	
Characteristic	N (%)
*Gender of participants (N=550)*	
Male	381 (69.3)
Female	169 (30.7)
*Age of participants (N=542)*	
Age≥50 y	184 (33.9)
Age<50 y	358 (66.1)
*Marital status (N=550)*	
Married	429 (78.0)
Separated	25 (4.6)
Divorced	33 (6.0)
Widowed	41 (7.4)
Cohabitating	6 (1.1)
Never married	16 (2.9)
*Educational level (N=550)*	
None	251 (45.6)
Primary school	274 (49.8)
Secondary school	9 (1.7)
Non-formal education	16 (2.9)
*Livelihood activities (N=550)*	
Small business owner	6 (1.1)
Subsistence farmer (cashew)	527 (95.8)
Unemployed (able to work)	2 (0.4)
Fishing	8 (1.4)
Unemployed (unable to work)	6 (1.1)
Other	1 (0.2)
*Monthly household expenditure (N=550)*	
<50 000 (US$22)	281 (51.1)
51 000 (US$22)−100 000 (US$43)	157 (28.5)
101 000 (US$43)−150 000 (US$65)	57 (10.4)
151 000 (US$65)−200 000 (US$87)	15 (2.7)
201 000 (US$87)−250 000 (US$109)	17 (3.1)
251 000 (US$109)−301 000 (US$131)	18 (3.3)
>301 000 (US$131)	5 (0.9)
*Relationship with children (N=550)*	
Parent	462 (84.0)
Grandparent	72 (13.1)
Uncle/aunt	3 (0.5)
Sibling	6 (1.1)
Other	7 (1.3)
*Main source of drinking water (N=550)*	
Surface water (river, dam, lake, pond)	317 (57.6)
Piped into yard/plot	1 (0.2)
Piped to neighbour	3 (0.6)
Public tap/standpipe	79 (14.3)
Tubewell/borehole	15 (2.7)
Protected well	40 (7.3)
Unprotected well	92 (16.7)
Rainwater	3 (0.6)
*Type of toilet facility (N=550)*	
Pit latrine with slab	11 (2.0)
Pit latrine without slap/open pit	522 (94.9)
No facility, bush, field	12 (2.2)
Other	5 (0.9)
*Type of cooking fuel (N=550)*	
Charcoal	1 (0.2)
Wood	549 (99.8)
*Place where cooking usually happens (N=550)*	
Separate room used as kitchen	82 (14.9)
Separate building	26 (4.7)
Outside	184 (33.5)
Inside	244 (44.4)
Other	14 (2.5)

### Diagnosis of (febrile) illness

About 89.3% (491) of household heads recalled one of their child's last fever episodes. It was generally detected by placing a hand on the forehead of the child (73.1% [359]) to see if they felt hot. A small minority had access to thermometers and could measure axial temperature (5.9% [29]). The diagnosis was made mostly by parents (72.1% [354]) and they did not seek advice from their friends/family regarding the illnesses (86.2% [423]) (Table 3).

**Table 3.** Last incidence of fever (Mtwara District Council, April–August 2020)

Last incidence of fever		
Characteristic	N (%)	95% CI
*Remember last incidence of fever/diarrhoea/bad cough*	491 (89.3)	(86.7–91.8)
*How fever was diagnosed (N=491)*		
Hand on head	359 (73.1)	(69.2–77.0)
Thermometer under arm	29 (5.9)	(3.9–7.9)
Thermometer—rectal	2 (0.4)	(0.0–1.0)
Thermometer—mouth	1 (0.2)	(0.0–0.6)
Other	100 (20.4)	(16.7–24.0)
*Who diagnosed fever (N=491)*		
Parent	354 (72.1)	(68.0–76.0)
Grandparent	48 (9.8)	(7.3–12.4)
Uncle/aunt	6 (1.2)	(0.4–2.2)
Sibling	7 (1.4)	(0.4–2.6)
Doctor	52 (10.6)	(7.7–13.2)
Other	24 (4.9)	(3.1–6.9)
*Advice from family/friends*		
Yes	68 (13.8)	(10.8–16.7)
No	424 (86.2)	(83.3–89.2)

Participants in the FGDs and IDIs recognised the severity of illnesses in children by assessing the progression of symptoms and observing their behaviour. Parents perceived a child as seriously ill when the child exhibited signs such as unusual tiredness, reduced playfulness, visible weakness and elevated body temperature. Parents would ask children who could communicate about their symptoms. Malaria was considered the most common problem in the community. Diseases such as diarrhoea and respiratory tract infections were equally reported as major health problems:

*The symptoms to help [assess] the child's illness has become severe are: the child having no desire to play, refusing to eat and other conditions, such as not being happy* (FGD-0802, 112-129).

*I recognise the severity of the illness because I am the head of the family. […] [W]hen he is sick, he will be sleeping all the time, and when you ask, he would say that the body, head, legs and other parts of the body are hurting, and they are weak. Therefore, I recognise that the illness is severe because when children are well, they go out and play* (IDI-082001, 50-62).

*The most common illnesses include malaria, cough and respiratory diseases and diarrhoea. These are the three most common illnesses that bothers people in this area* (KII-HC08007, 26-36).

### Care seeking

Four-fifths (79.0% [388]) of participants sought medical care when their children fell sick; 72.7% (282) went to healthcare facilities. The decision to seek medical care was based on severity of illness (95.4% [370]), which echoed the care seeking for older people, of whom 60% (330) were sent to the hospital only when they had severe illnesses (Table 4).

**Table 4.** Decision to seek healthcare (Mtwara District Council, April–August 2020)

Decision to seek care		
Characteristic	N (%)	95% CI
*Action taken after first diagnosis (N=491)*		
Seek care	388 (79.0)	(75.4–82.7)
Wait	23 (4.7)	(3.1–6.7)
Treat at home	80 (16.3)	(12.8–19.6)
*If sought care, where (N=388)*		
Health facilities	282 (72.7)	(68.3–77.1)
Drug store	80 (20.6)	(16.8–24.5)
Herbalist	1 (0.3)	(0.0–0.8)
Other	25 (6.4)	(4.1–9.0)
*Time taken to decide seeking care*		
Decision seeking care≤12 h	248 (63.9)	(59.3–68.8)
Decision seeking care>12 h	140 (36.1)	(31.2–40.7)
*Time taken to travel to health facility*		
Travel time≤1 h	137 (78.3)	(72.6–84.6)
Travel time>1 h	38 (21.7)	(15.4–27.4)
*Treatment costs (N=282)*		
<10 000 (US$4.4)	145 (51.4)	
11 000 (US$4.8)–50 000 (US$21.7)	58 (20.6)	
51 000 (US$22.2)–100 000 (US$43.5)	6 (2.1)	
>101 000 (US$43.9)	2 (0.7)	
Did not incur any cost	61 (21.6)	
We use health insurance	10 (3.6)	
*When would it be good for a person aged >50 y to go to local hospital? (multiple response)* High fever	333 (60.6)	
Bad cough	21 (3.8)	
Breathless	13 (2.4)	
Fear of dying	23 (4.2)	
Severity of diseases	330 (60)	

**Figure 1:**
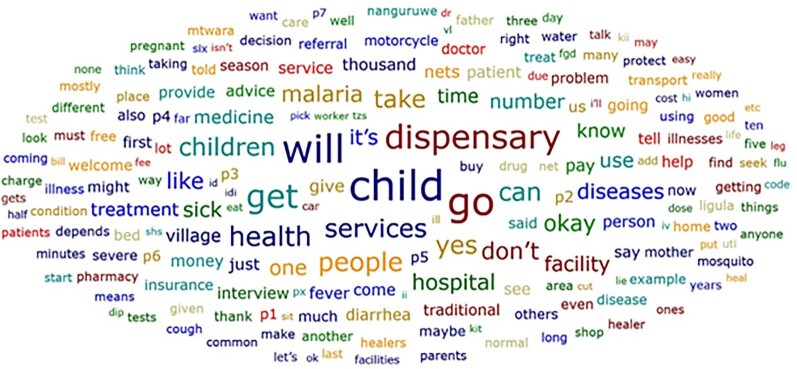
Words crowd from all interviews and FGDs (Mtwara District Council, April–August 2020). In this word crowd, the words in large font are those that were most used during the interviews. This is an indication that children were most likely to be brought to the health facilities (dispensary) when they became ill.

Timeliness in seeking care was assessed by the number of hours that had elapsed between identifying the child's illness and seeking care. One-third (36.1% [140]) of the household heads took >12 h to decide on seeking healthcare (Table 4). Parents often sought treatment at the nearby dispensary (Figure 1):

*If my child or the neighbour's child has fever, the first thing is to go at the dispensary to get served there. But there at the dispensary, after [receiving care], you can check the fever and see how it's going. If you see no improvement, you can go to another place, like the regional hospital* (FGD-0812, 279-279).

*When the children get this problem, most of the time, we take them to the dispensary, for example here in Magomeni our nearest dispensary is at Tangazo* (IDI-042010, 62-63).

*They go to the dispensary, talk to the doctor and the doctor will direct them in terms of what to do* (KII-CH08009, 288-307).

There was no significant association of health-seeking behaviour with gender, education level or household expenditure among participants (Table 5).

**Table 5.** Action taken vs gender, relationship with children, education level, monthly household expenditure (Mtwara District Council, April—August 2020)

Action taken after realising that the child is sick								
Variable	Seek care		Treat at home		Wait		Total	p
*Gender (N=388)*	N	%	N	%	N	%		
Female	108	72%	31	21%	10	7%	149	0.055
Male	281	82%	49	14%	13	4%	343	
*Relationship with children aged <18 y*								
Parent	332	80%	65	16%	17	4%	414	0.111
Grandparent	45	70%	13	20%	6	9%	64	
Uncle/aunt	1	33%	2	67%	0	0%	3	
Sibling	5	100%	0	0%	0	0%	5	
Other	5	100%	0	0%	0	0%	5	
*Total*	*388*	79%	*80*	16%	*23*	5%	491	
								
*Education level (head of household)*								
None	167	75%	41	18%	14	6%	222	0.516
Primary	202	82%	35	14%	9	4%	246	
Secondary	7	88%	1	13%	0	0%	8	
Non-formal education	12	80%	3	20%	0	0%	15	
*Total*	*388*	79%	*80*	16%	*23*	5%	491	
								
*Monthly household expenditure*								
<50 000 (US$22)	193	79%	37	15%	14	6%	244	0.646
51 000 ($22)−100 000 (US$43)	112	79%	23	16%	6	4%	141	
101 000 (US$43)−150 000 (US$65)	41	77%	9	17%	3	6%	53	
151 000 (US$65)−200 000 (US$87)	13	93%	1	7%	0	0%	14	
201 000 (US$87)−250 000 (US$109)	10	63%	6	38%	0	0%	16	
251 000 (US$109)−301 000 (US$131)	15	83%	3	17%	0	0%	18	
>301 000 (US$131)	4	80%	1	20%	0	0%	5	
*Total*	*388*	79%	*80*	16%	*23*	5%	491	

Although participants described initially seeking care at healthcare facilities, insights from IDIs, KIIs and FGDs revealed that participants opted to treat at home using traditional herbs or purchase medication from drug stores before eventually seeking care at dispensaries:

*When my children get sick, first, I go to the pharmacy, and if the pharmacy provider tells me to go to the dispensary, then I will go* (IDI-041011, 262-266).

*Also, if you don't have money you can decide to take leaves that cure the disease. For example, neem leaves (mwarubaini): you boil [them] and leave it for some time, then later you drink it and use it to treat the body* (IDI-082001, 82-86).

This kind of health-seeking behaviour was seen by some as contributing to delays in seeking care, promoting the progression of illnesses:

*They don't bring the child to the hospital early; they bring the child to the hospital when the illness is severe. However, there are few who understand and bring the children early to the hospital, but most of them bring them when the condition of the illness is worse* (KII-CH08013, 33-33).

*But, most of the time, we usually don't take the child to the hospital on the first day we notice that s/he is unwell. We just buy ‘Sheladol’* (a painkiller) *for them. We take the child to the hospital on the second day after his/her condition worsens* (FGD-0814, 77-114).

Traditional medicine or healers were sought for illnesses perceived as untreatable at the hospital. When family members were repeatedly brought to the hospital with a complaint and it was not cured, the community members perceived that the disease could only be treated by a traditional healer:

*There are diseases, [and] even if you take the child to the hospital, the child will not get any treatment. For example, when you went to the market and suddenly the leg starts hurting and swelling then, my friend you cannot go to the hospital you must go to spiritual or traditional healers, so that you can get treatment. It is true when you go there, they will tell you that someone [bewitched you] on the way and when they give you medicine for three to four days, the legs will improve* (IDI-082001, 122-125).

Participants were concerned about affordability of healthcare (61.4% [338]). Study participants made cash payments at the healthcare facilities, with the overall treatment cost being <10 000Tsh (US$4.3) for those who sought care (51.4% [145]), equivalent to one-quarter of their monthly expenditure. Around one in five respondents (20.6% [58]) paid 11 000 (US$4.8)–50 000Tsh (US$21.7), while 21.6% (60) did not incur any cost because healthcare was provided free for children aged <5 y; a very small percentage incurred >50 000Tsh (US$21.7) for treatment. Only 10 (3.6%) households who utilised healthcare had health insurance at the time of the survey (Table 4):

*Most of us are not financially well-off enough to pay for health insurance, but a few people are using health insurance. In our dispensary they have outlined that, from six years and above, you need the average of nine thousand shillings to cover the costs of all the services that you need at [the] dispensary* (FGD-0804, 178-182).

*Yes, health insurance. Those who have money, they pay for the insurance and get an insurance card, which can be used by six people in the household* (IDI-81007, 121-122).

Respondents described how children aged <5 y were not required to pay for healthcare services. When children aged ≥5 y or adults became ill then the cost of treatment was perceived to be a major deterrent to seeking healthcare:

*You pay depending on the patient because, below five years [of age], the patient is treated for free. But, for six years and above, a test for malaria costs 2000 shillings; seeing the doctor is 1000 shillings; and testing for a urinary tract infection is 4000 shillings. Therefore, you must pay for any lab test* (FGD-0804, 153-163).

The clinical officer in one of the health facilities explained how they categorised patients. Treatment was free for under-five children, TB and HIV patients and pregnant women. He further explained that the medicines were free for special conditions, such as epilepsy and asthma. Those who did not have health insurance had to pay for health services. There were two types of health insurance available, National Health Insurance Fund and Improved Community Health Fund. Although healthcare for under-five children was provided free, because of stock-outs, sometimes parents were obliged to buy medicines from drug stores.

Respondents described the distance to the healthcare facility as a major determinant of health-seeking behaviour. Travel time and expenses influenced care seeking, yet health facilities were reported to be mostly within 1 h on foot from respondents’ homes (78.3% [138]):

*…with motorcycle, it is half an hour to 40 minutes and the fare is 7000 to 10 000TSh for a return trip* (KII-CH04004, 268-309).

Perceived quality of care influenced healthcare seeking. Respondents were concerned with the number of staff at facilities and they described how insufficient staff numbers had implications for the care delivered. Healthcare workers acknowledged deficits in staffing and medical supplies necessary for providing quality care:

*You might find a nurse is attending a malaria patient and at the same time there is a woman who is due to deliver. It is very difficult* (FGD-0804, 411-422).

*For example, in our dispensary, it would be better if they improve by increasing medical supplies because most of the time we are told that there is no medicine* (FGD-0415, 688-713).

Apart from understaffing and a shortage of medical supplies, undesirable interactions between clients and service providers were said to affect healthcare utilisation. Some service providers were alleged to be untrustworthy because they asked for bribes from patients. Lack of confidence on the part of either party led to underutilisation of healthcare:

*A child won't get tested without cash. If you don't give 2000Tsh, a child would be sent back home, or they will collect her/his registration/clinic card until you pay. The healthcare provider will ignore you if you don't give her/him something. This should be looked at* (FGD-814, 423-436).

## Discussion

In rural Mtwara, Tanzania, seeking care for childhood illnesses depended on personal factors, such as the perceived severity of illness, and benefits of early treatment, as well as institutional factors, such as accessibility and the cost of health services.^15^ At the community level, healthcare seeking was influenced by sociocultural dynamics, such as beliefs related to specific illnesses, including perceived severity and the perceived competence of healthcare services.^4^ Respondents generally sought care for illnesses with severe signs and symptoms.^16^ Seeking care at health facilities was, however, often delayed until symptoms were severe.^15^ Lack of urgency has also been established as an independent factor affecting treatment-seeking behaviour.^17^

Institutional factors, such as the availability, accessibility, affordability and acceptability of healthcare, had a significant impact on healthcare seeking. These findings align well with the WHO’s description of how institutional factors affect the coverage and quality of care.^18^ Availability refers to the existence of obtainable health services and medications.^19^ Most of the health facilities in this study were within 1-h walking distance, which suggests reasonable coverage,^20^ although this might not necessarily mean good access and quality care. Understaffing and stock-outs influenced care seeking, regardless of accessibility.^21^

Although respondents recognised the need to seek care when a child displayed symptoms, uncertainty and inconsistency of services in facilities, particularly anticipated long waits and medicines stockouts, affected their responses. As a result, parents may anticipate and thus seek treatment at private drug shops, ultimately undermining the achievement of universal health coverage, even when risk pooling through health insurance is introduced. A recent review also highlighted the challenges in accessing healthcare for community members, suggesting the need to enhance community-based health services in the rural communities of Tanzania.^22^

Care seeking was affected by the availability of funds to pay for transport and healthcare costs at facilities. Indeed, in many settings, a lack of access to cash to cover expenses often affects treatment-seeking behaviour.^23^ Most respondents explained the absence of health insurance and attributed this to financial cosntraints. Even if they had health insurance, they had to make some cash payments because of occasional health facility stock-outs, which forced them to purchase medication from private drug stores.^24^ Out-of-pocket payments for healthcare are anathema to the idea of universal coverage of healthcare and significantly affect the sustainable development goals.^25,26^ Tanzania established a low-cost health insurance system (Community Health Fund) in 2001 to provide the poor rural community with access to a prepaid system of healthcare. This health insurance scheme costs 30 000Tsh (US$13) per year for a family of six people. By 2018, 2.1 million households and 12.6 million beneficiaries were enrolled.^27^ Nonetheless, the implementation of this health insurance scheme has faced challenges and its uptake is still limited. Respondents regarded enrollment in the Community Health Fund as expensive and unaffordable.^21^

### Strengths and limitations

Most of the data in this study were collected from the household heads and we did not triangulate the information with the health facilities, drug stores and traditional healers. This potentially gives a one-sided perspective on care seeking. The study may have incurred recall and social desirability biases. Aligning with the original analysis plan, we did not model the patterns of delayed care seeking, including factors and their magnitude, in affecting outcomes for specific children. Future research can incorporate regression models to explore factors affecting care-seeking behaviour.

### Conclusion

Improving access to healthcare facilities and ensuring the availability of high-quality services is a global priority. This study of care seeking for childhood illnesses in Mtwara, Tanzania, highlighted the relevance of institutional factors along with other influences. Hence, increasing access without improving quality of services may not promote greater utilisation of health facilities. In this setting, future research is needed to assess the impact of health insurance schemes on care quality and care seeking. Furthermore, data collection took place before the Star Homes project and follow-up research should assess the impact of its implementation on care seeking in participating communities.

## Data Availability

The data underlying this article will be shared upon reasonable request to the corresponding author; this is due to protection of the privacy of those individuals who participated in this study.

## References

[1] Word Health Organization . World Malaria Report 2021. World Health Organization. 2021. Available from https://www.who.int/teams/global-malaria-programme/reports/world-malaria-report-2021 [accessed August 2023].

[2] Demissie GD, Yeshaw Y, Aleminew W et al. Diarrhea and associated factors among under five children in sub-Saharan Africa: evidence from demographic and health surveys of 34 sub-Saharan countries. PLoS One. 2021;16(9 September):1–13. 10.1371/journal.pone.0257522 PMC8452002 34543347

[3] Seidu AA, Dickson KS, Ahinkorah BO et al. Prevalence and determinants of acute lower respiratory infections among children under-five years in sub–Saharan Africa: evidence from demographic and health surveys. SSM-Popul Heal. 2019;8:100443. 10.1016/j.ssmph.2019.100443 PMC6614699 31334326

[4] Ngwenya N, Nkosi B, McHunu LS et al. Behavioural and socio-ecological factors that influence access and utilisation of health services by young people living in rural KwaZulu-Natal, South Africa: implications for intervention. PLoS One. 2020;15(4):e0231080. 32287276 10.1371/journal.pone.0231080 PMC7156071

[5] Osborn D, Cutter A, Ullah F. Universal sustainable development goals. Understanding the transformational challenge for developed countries. Report of a study by stakeholder forum. 2015;2(1):1–25. Available from https://sustainabledevelopment.un.org/content/documents/733FutureWeWant.pdf [accessed August 2023].

[6] Falchetta G, Hammad AT, Shayegh S. Planning universal accessibility to public health care in sub-Saharan Africa. Proc Natl Acad Sci USA. 2020;117(50):31760–9. 33257557 10.1073/pnas.2009172117 PMC7749323

[7] Abegaz NT, Berhe H, Gebretekle GB. Mothers/caregivers healthcare seeking behavior towards childhood illness in selected health centers in Addis Ababa, Ethiopia: a facility-based cross-sectional study. BMC Pediatr. 2019;19(1):1–9. 31269920 10.1186/s12887-019-1588-2 PMC6607537

[8] Clarke SE, Rowley J, Bøgh C et al. Home treatment of “malaria” in children in rural Gambia is uncommon. Trop Med Int Heal. 2003;8(10):884–94. 10.1046/j.1365-3156.2003.01095.x 14516299

[9] Mitiku I, Assefa A. Caregivers’ perception of malaria and treatment-seeking behaviour for under five children in Mandura District, West Ethiopia: a cross-sectional study. Malar J. 2017;16(1):1–10. 28390423 10.1186/s12936-017-1798-8 PMC5385040

[10] Kunnuji M, Wammanda RD, Ojogun TO et al. Health beliefs and (timely) use of facility ‑ based care for under ‑ five children : lessons from the qualitative component of Nigeria’ s 2019 VASA. BMC Public Health. 2022;22(1):1–3. 35484514 10.1186/s12889-022-13238-1 PMC9047270

[11] Mshamu S, Mmbando A, Meta J et al. Assessing the impact of a novel house design on the incidence of malaria in children in rural Africa: study protocol for a household-cluster randomized controlled superiority trial. Trials. 2022;23(1):1–14. 35725486 10.1186/s13063-022-06461-z PMC9207857

[12] Mshamu S, Peerawaranun P, Kahabuka C et al. Old age is associated with decreased wealth in rural villages in Mtwara, Tanzania: findings from a cross-sectional survey. Trop Med Int Heal. 2020;25(12):1441–9. 10.1111/tmi.13496 PMC7756872 32985048

[13] Tanzania National Beareu of statistics . Administrative Units Population Distribution Report. National Population and House Census of Tanzania. National Bureau of Statistics, Dar es Salaam, Tanzania. 2022. Available from https://www.nbs.go.tz/index.php/en/census-surveys/population-and-housing-census/852-2022-population-and-housing-census-administrative-units-population-distribution-and-age-sex-reports [accessed August 2023].

[14] Mremi IR, Mbise M, Chaula J. Distribution of primary health care facilities in Mtwara District, Tanzania: availability and accessibility of services. Tanzan J Health Res. 2018;20(4).

[15] Simieneh MM, Mengistu MY, Gelagay AA et al. Mothers’ health care seeking behavior and associated factors for common childhood illnesses, Northwest Ethiopia: community based cross-sectional study. BMC Health Serv Res. 2019;19(1):1–7. 30674309 10.1186/s12913-019-3897-4 PMC6343298

[16] Fikire Id A, Ayele G, Haftu D. Determinants of delay in care seeking for diarrheal diseases among mothers/caregivers with under-five children in public health facilities of Arba Minch town, southern Ethiopia; 2019. PLoS One. 2020;15(2):e0228558. 32053615 10.1371/journal.pone.0228558 PMC7018063

[17] Marahatta SB, Yadav RK, Giri D et al. Barriers in the access, diagnosis and treatment completion for tuberculosis patients in central and western Nepal: a qualitative study among patients, community members and health care workers. PLoS One. 2020;15(1):1–18. 10.1371/journal.pone.0227293 PMC6961875 31940375

[18] Word Health Organization . Global Strategy on human Resources for Health: Workforce 2030. WHO. 2016;64. Available from https://www.who.int/hrh/resources/global_strategy_workforce2030_14_print.pdf?ua=1 [accessed August 2023].

[19] Randall T. Malaria-associated health-seeking behaviour among the Jola of the Gambia, West Africa. Anthropol Action. 2013;20(1):4–17.

[20] Jaca A, Malinga T, Iwu-Jaja CJ et al. Strengthening the health system as a strategy to achieving a universal health coverage in underprivileged communities in Africa: a scoping review. Int J Environ Res Public Health. 2022;19(1):587.21. 35010844 10.3390/ijerph19010587 PMC8744844

[21] Macha J, Kuwawenaruwa A, Makawia S et al. Determinants of community health fund membership in Tanzania: a mixed methods analysis. BMC Health Serv Res. 2014;14(1):1–1. 25411021 10.1186/s12913-014-0538-9 PMC4246628

[22] Adhikari B, Bayo M, Peto TJ et al. Comparing the roles of community health workers for malaria control and elimination in Cambodia and Tanzania. BMJ Glob Heal. 2023;8(12):1–9. 10.1136/bmjgh-2023-013593 PMC10729139 38070880

[23] Mburu CM, Bukachi SA, Shilabukha K et al. Determinants of treatment-seeking behavior during self-reported febrile illness episodes using the socio-ecological model in Kilombero District. Tanzania BMC Public Health. 2021;21(1):1–11. 34090402 10.1186/s12889-021-11027-w PMC8180143

[24] DeVoe JE, Baez A, Angier H et al. Insurance + access ≠ health care: typology of barriers to health care access for low-income families. Ann Fam Med. 2007;5(6):511–8. 18025488 10.1370/afm.748 PMC2094032

[25] Dye C. Expanded health systems for sustainable development. Science. 2018;359(6382):1337–9. 29567697 10.1126/science.aaq1081

[26] McIntyre D, Garshong B, Mtei G et al. Beyond fragmentation and towards universal coverage: insights from Ghana, South Africa and the United Republic of Tanzania. Bull World Health Organ. 2008;86(11):871–6. 19030693 10.2471/BLT.08.053413 PMC2649570

[27] Lee B, Tarimo K, Dutta A. Tanzania's improved community health fund: an analysis of scale-up plans and design. Health Policy Plus brief. 2018. Available from http://www.healthpolicyplus.com/ns/pubs/10259-10469_TanzaniaiCHFScaleUpbrief.pdf [accessed August 2023].

